# Control of directional change after mechanical stimulation in Drosophila

**DOI:** 10.1186/1756-6606-5-39

**Published:** 2012-10-29

**Authors:** Yating Zhou, Scott Cameron, Wen-Tzu Chang, Yong Rao

**Affiliations:** 1 McGill Centre for Research in Neuroscience, McGill University Health Centre, 1650 Cedar Avenue, Montreal, Quebec H3G 1A4, Canada; 2 Department of Biology, McGill University Health Centre, 1650 Cedar Avenue, Montreal, Quebec, H3G 1A4, Canada; 3 Department of Neurology and Neurosurgery, McGill University Health Centre, 1650 Cedar Avenue, Montreal, Quebec, H3G 1A4, Canada; 4 Department of Medicine, McGill University Health Centre, 1650 Cedar Avenue, Montreal, Quebec, H3G 1A4, Canada; 5 Centre for Research in Neuroscience, McGill University Health Centre, Room L7-136, 1650 Cedar Avenue, Montreal, Quebec, H3G 1A4, Canada

## Abstract

**Background:**

Proper adjustment of moving direction after external mechanical stimulation is essential for animals to avoid danger (e.g. predators), and thus is vital for survival. This process involves sensory inputs, central processing and motor outputs. Recent studies have made considerable progress in identifying mechanosensitive neurons and mechanosensation receptor proteins. Our understandings of molecular and cellular mechanisms that link mechanosensation with the changes in moving direction, however, remain limited.

**Results:**

In this study, we investigate the control of movement adjustment in Drosophila. In response to gentle touch at the anterior segments, Drosophila larvae reorient and select a new direction for forward movement. The extent of change in moving direction is correlated with the intensity of tactile stimuli. Sensation of gentle touch requires chordotonal organs and class IV da neurons. Genetic analysis indicates an important role for the evolutionarily conserved immunoglobulin (Ig) superfamily protein Turtle (Tutl) to regulate touch-initiated directional change. Tutl is required specifically in post-mitotic neurons at larval stage after the completion of embryonic development. Circuit breaking analysis identified a small subset of Tutl-positive neurons that are involved in the adjustment of moving direction.

**Conclusion:**

We identify Tutl and a small subset of CNS neurons in modulating directional change in response to gentle touch. This study presents an excellent starting point for further dissection of molecular and cellular mechanisms controlling directional adjustment after mechanical stimulation.

## Background

Proper adjustment of moving direction is essential for animals to forage and to escape from predation. Animals use cues such as light, odor, temperature and mechanical stimuli to make their movement decisions [1]. The focus of this study is to understand the mechanisms that regulate the adjustment of moving direction after gentle touch.

Reorientation of movement after mechanical stimulation requires activation of mechanosensitive neurons, the integration and processing of information in the central nervous system (CNS), and motor outputs (as reviewed by [2,3]). Recent studies in genetic model systems such as Drosophila and C. elegans have shed light on molecular mechanisms underlying the activation of mechanosensitive neurons [4,5]. For instance, genetic screen in C. elegans led to the identification of mec-4 and mec-10, which encode mechanotransducers (i.e. DEG/ENaC channels) [6]. Genetic dissection of mechanosensation in Drosophila also identified NompC, a member of the TRP channel family, as a mechanotransducer [7,8]. However, less is known about how the information from mechanosensory neurons is processed in the CNS for animals to adjust their moving direction.

Drosophila is an excellent model system for understanding molecular and cellular mechanisms underlying directional change after mechanical stimulation. The anatomy and development of mechanosensory organs in Drosophila have been well studied [4,9]. Molecules important for mechanotransduction have been identified in Drosophila, such as mechanotransducers Pickpocket [10], Piezo [11] and NompC [7,8], as well as other proteins that are required for maintaining the structural integrity of mechanosensitive neurons (e.g. NompA) [12]. Recent development of sophisticated techniques that allow spatial and temporal manipulation of circuit activity in living flies (e.g. [13-15]), greatly facilitates the study of neuronal circuitry underlying specific behaviors.

In this study, we investigate the mechanisms that regulate the adjustment of moving direction by Drosophila larva in response to gentle touch. We examined the modulation of directional change by gender difference, the intensity of tactile stimuli, and the nociceptive pathway. We also performed genetic analyses to gain insights into underlying molecular and cellular mechanisms. We show that the adjustment of moving direction after gentle touch requires the *turtle* (*tutl)* gene, which encodes an evolutionarily conserved Ig-superfamily transmembrane protein. Our results also implicate a role for a small subset of Tutl-positive neurons in modulating the pattern of directional change.

## Results

### Larvae adjust moving direction after gentle touch

**Figure 1:**
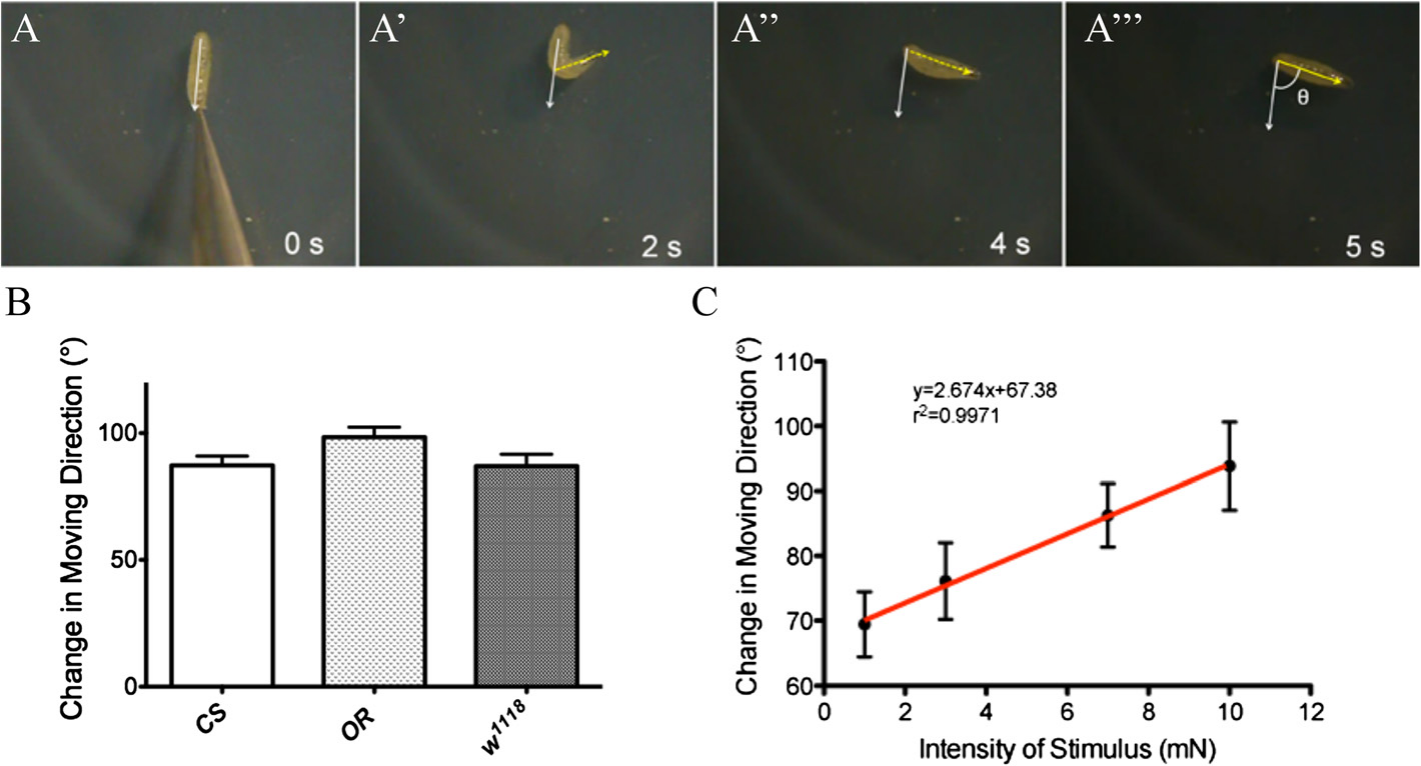
**Wild-type *Drosophila *larvae display stereotyped navigational pattern in response to gentle touch.** (**A**-**A**”’) Time course of navigational pattern of wild-type 3^rd^-instar larvae in response to tactile stimuli at anterior segments. “θ” refers to the angle between original direction and reoriented direction of forward movements. The reoriented direction was measured when a larva finished one peristalsis after resuming its forward locomotion. (**B**) Quantification of larval navigational pattern in response to tactile stimuli. *Canton-S* (*CS*) (n=24), *Oregon-R* (*OR*) (n=34) and *w-1118* larvae (n= 28) showed similar navigational pattern in response to tactile stimulus (7 mN). P>0.05 (one-way ANOVA). (**C**) Linear regression relationship between the extent of directional changes (°) and the intensity of tactile stimulus (mN). The best-fit line is shown in red. Number of larvae tested: 1 mN, n=28; 3 mN, n=27; 7 mN, n=27; 10 mN, n=26. Error bars represent SEM.

Wild-type *Drosophila* larvae display stereotyped responses to gentle touch at the anterior part including head and thoracic segments [7]. A typical larval response to a tactile stimulus during normal forward locomotion (Figure 1A) consists of quick withdrawal by contracting their anterior segments, brief hesitation and one or more exploratory head swings (Figure 1A’), reorientation of entire body (Figure 1A”), and resuming forward movements in a new direction (Figure 1A”’). In some cases, one or more complete waves of reverse contractions are made before selecting a new direction for forward movement. Such change in moving direction is necessary for a larva to avoid re-encountering the stimuli.

To quantify the data, we measured the angle (“θ” in Figure 1A”’) between the directions of original and reoriented forward movement. Similar navigational pattern was observed in *Canton-S* (*CS*), *Oregon-R* (*OR*), and *w1118* larvae (Figure 1B). We also found that male and female larvae showed similar navigational pattern in response to gentle touch (data not shown). No significant difference in withdrawal response (data not shown), responding time (data not shown), or selection of new moving direction (data not shown), was observed between male and female larvae.

### The intensity of tactile stimuli affects navigational pattern

To determine if the level of sensory inputs affects navigational pattern, we applied different intensities of tactile stimuli (i.e. 1 mN, 3 mN, 7 mN and 10 mN) with calibrated filaments to the anterior segments (see Methods). Interestingly, we found that the extent of directional change after tactile stimuli was correlated with the intensity of stimuli (Figure 1C). In response to an increase in intensity from 1 mN to 10 mN, the average change in forward movement direction was increased from 69.4° to 93.8° (Figure 1C). The data fit a linear regression model, indicating a significant correlation between the intensity of stimulus and directional change (Figure 1C).

### Painless-mediated nociceptive pathway was not involved in regulating directional change after gentle touch

Previous studies in Drosophila suggest that the mechanisms of sensing gentle touch are different from that of nociception [7,10,11,16]. If so, one would predict that directional change after gentle touch should not require the activation of nociceptive pathway. To test this, we examined the response of *painless (pain)* mutants to gentle touch. *pain* encodes a member of TRPN channels. *pain* is expressed in multidendritic neurons (md) and chordotonal organs, and is required for both mechanical and thermal nociception [16].

**Figure 2:**
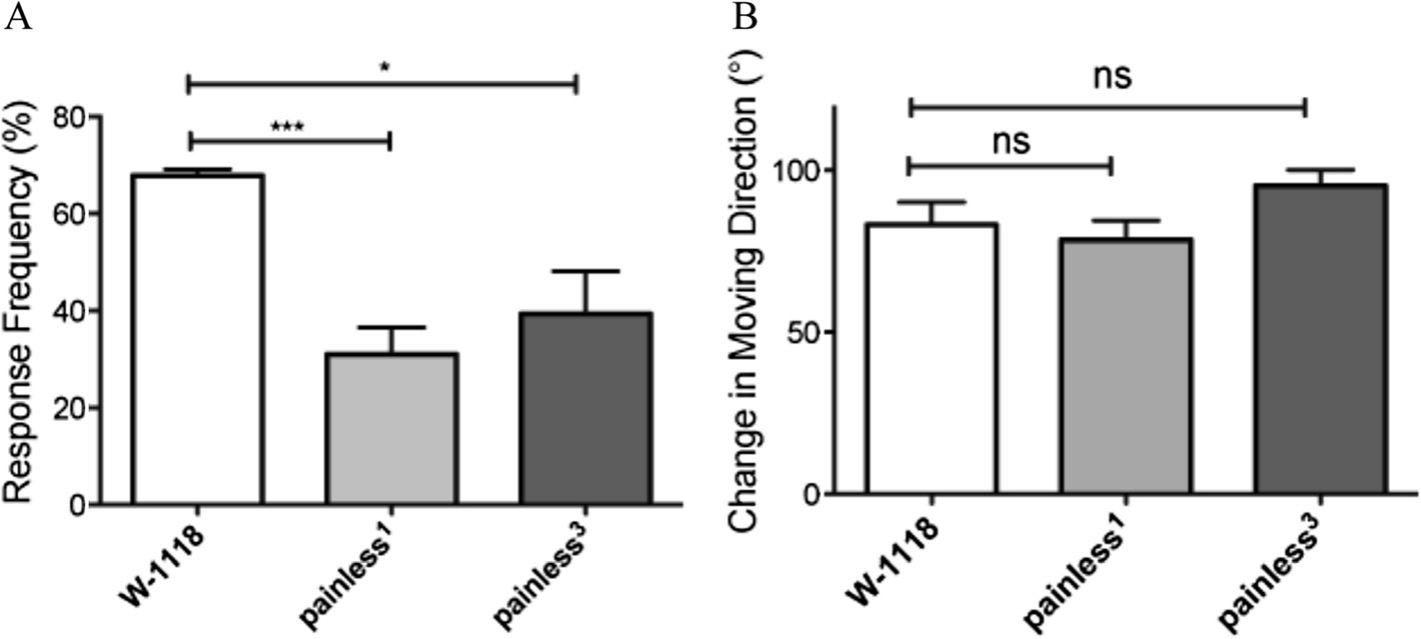
***Painless *mutant larvae displayed normal navigational pattern in response to gentle touch.** (**A**) Nociceptive behaviors of wild-type and *pain* mutants in response to noxious mechanical stimuli (50 mN) were examined. Response frequency (i.e. the percentage of larvae that showed complete rolling-over behaviors) of larvae was examined (three trials). Number of larvae tested: *w-1118*, n=83; *pain*^*1*^, n=74; *pain*^*3*^, n=91. ^*^p < 0.05, ^***^p<0.005, t-test. (**B)** Navigational pattern of *pain* mutant larvae in response to tactile stimuli was examined. Number of larvae tested: *w-1118*, n=21; *pain*^*1*^, n=26; *pain*^*3*^, n=26. p>0.1 for t-test and one-way ANOVA test. Error bars represent SEM.

Consistent with a previous report [16], both *pain*^*1*^ and *pain*^*3*^ mutant larvae showed significant defects in nociception (Figure 2A). In response to a noxious mechanical stimulus of 50 mN (Von Frey fibers) on the dorsal midline, most wild-type larvae displayed a nocifensive escape behavior by rotating around their long body axis (Figure 2A). In contrast, both *pain*^*1*^ and *pain*^*3*^ mutant larvae showed a significant reduction in the response frequency.

We then examined navigational pattern of *pain*^*1*^ and *pain*^*3*^ mutant larvae in response to gentle touch. Compared to wild type, no significant difference in navigational behaviors was observed in *pain*^*1*^ and *pain*^*3*^ mutant larvae (Figure 2B). This result suggests strongly that directional adjustment after gentle touch involves a Pain-independent pathway.

### Sensation of gentle touch requires class IV da neurons and chordotonal organs

Previous studies suggest that chordotonal organs are involved in sensing gentle touch in larvae [17]. To determine the potential role of chordotonal organs in navigational pattern after gentle touch, we examined the effect of blocking synaptic transmission from chordotonal organs by expressing a temperature-sensitive form of *shibire* (*shi*^ts^) that encodes the fly homolog of dynamin. The expression of *shi*^ts^ was under control of the chordotonal-specific driver iav-GAL4 [18]. This allows the blockage of synaptic transmission in targeted neurons at restrictive temperature [13].

**Figure 3:**
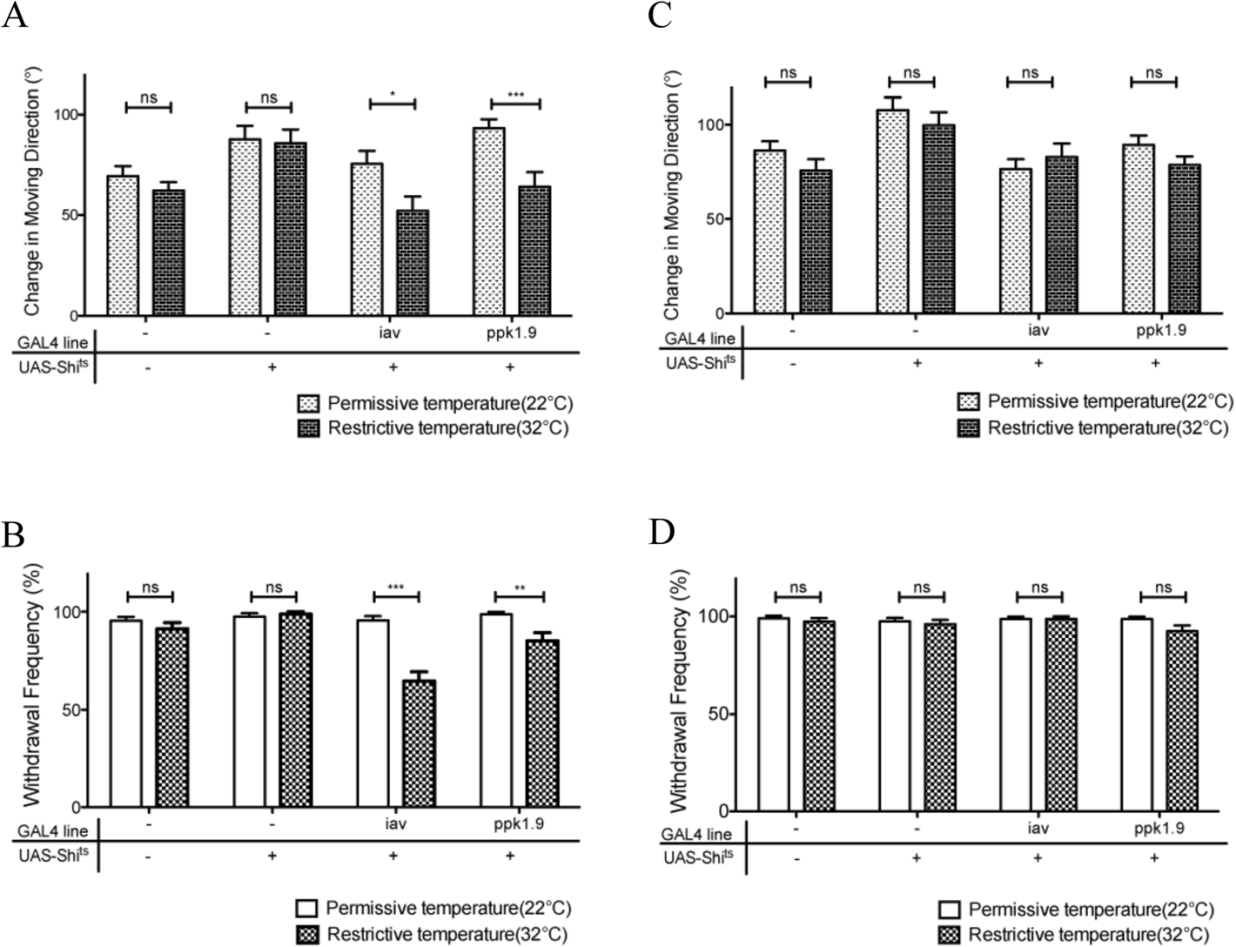
**Chordotonal organs and class IV da neurons were involved in sensing gentle touch.** UAS-*shi*^ts^ were expressed under control of chordotonal-organ-specific driver iav-GAL4 or class IV da neuron-specific driver ppk1.9-GAL4. After gentle touch, larval navigational pattern (**A**, **C**) and withdrawal response (**B**, **D**) were examined. The performance of larvae at restrictive temperature (32°C, dark bars) was compared to that of same-genotype larvae at permissive temperature (22°C, light bars). ^*^p<0.05, ^**^p<0.01, ^***^p<0.005, “ns” indicates p>0.05, t-test. (A) Navigational pattern of 3^rd^-instar larvae in response to 1 mN stimulus. (**B** ) Withdrawal response of larvae after gentle touch of 1 mN. Larvae tested in A and B: *W-1118*, n=20; UAS-*shi*^ts^, n=20; iav-GAL4 + UAS-*shi*^ts^, n=26; ppk1.9-GAL4 + UAS-*shi*^ts^, n=20. (**C**) Navigational pattern of 3^rd^-instar larvae in response to 7 mN stimulus. (**D**) Withdrawal response of larvae after gentle touch of 7 mN. Larvae tested in C and D: *W-1118*, n=19; UAS-*shi*^ts^, n=19; iav-GAL4 + UAS-*shi*^ts^, n=20; ppk1.9-GAL4 + UAS-*shi*^ts^, n=20. Note that expression of *shi*^ts^ driven by iav-GAL4 or ppk1.9-GAL4 at restrictive temperature significantly affected navigational pattern and withdrawal response in response to 1 mN stimuli. However, no significant effects were observed when the intensity was increased to 7 mN. Error bars represent SEM.

A shift from permissive temperature (i.e. 22°C) to restrictive temperature (i.e. 32°C ) did not affect navigational pattern by wild-type larvae after gentle touch of 1 mN or 7 mN intensity (Figure 3A and C). At restrictive temperature, expression of temperature-sensitive *shi* in all peripheral sensory neurons under control of the SN (5–40)-GAL4 driver [19], induced larval paralysis (100%, n=16), consistent with circuit breaking activity of *shi*^ts^ reported previously [13]. Interestingly, we found that blocking synaptic transmission in chordotonal organs significantly affected navigational pattern, as many larvae failed to change their moving direction in response to 1 mN tactile stimuli (Figure 3A). To test if this effect on navigational behavior was due to a reduction in mechanosensation, we examined withdrawal response that occurs immediately after tactile stimuli prior to reorientation of forward movement. Indeed, we found that many larvae did not withdraw from touch of 1 mN intensity (Figure 3B), consistent with a role for chordotonal organs in sensing gentle touch. When the intensity of stimulus was increased to 7 mN, however, navigational pattern and withdrawal response of larvae in which sensory inputs from chordotonal organs were blocked, occurred similarly as that in wild type (Figure 3C and D). This result suggests the involvement of other types of sensory neurons in sensing gentle touch.

Previous studies report that class IV dendritic arborization (da) sensory neurons mediate mechanotransduction in response to noxious mechanical (>30 mN) stimuli [10,20]. To determine if class IV da neurons also play a role in sensing gentle touch, we examined navigational pattern in larvae in which sensory inputs from class IV da neurons were blocked by expressing *shi*^ts^ under control of class-IV-da-specific driver pickpocket 1.9-GAL4 (ppk-GAL4) [21] at restrictive temperature. We found that blocking class IV da neurons also significantly affected withdrawal response and subsequent directional change after 1 mN stimulus (Figure 3A and B), while no effect was observed after 7 mN stimulus (Figure 3C and D). Together, these results suggest strongly that class IV da neurons and chordotonal organs are involved in sensing gentle touch.

### Mutations in *tutl* affected larval navigational pattern after gentle touch

To understand molecular and cellular mechanisms that modulate directional change after gentle touch, it is necessary to elucidate molecular networks that regulate the formation and function of neuronal circuitry involved. In a search for genes controlling larval navigational pattern, we found that mutations in the *turtle* (*tutl*) gene caused a severe defect in adjusting moving direction after gentle touch. *tutl* encodes an evolutionarily conserved Ig-superfamily transmembrane protein [22]. It is highly homologous to Dasm1 in mice and IgSF9 in humans [22-24], whose function in mammals remains unknown.

**Figure 4:**
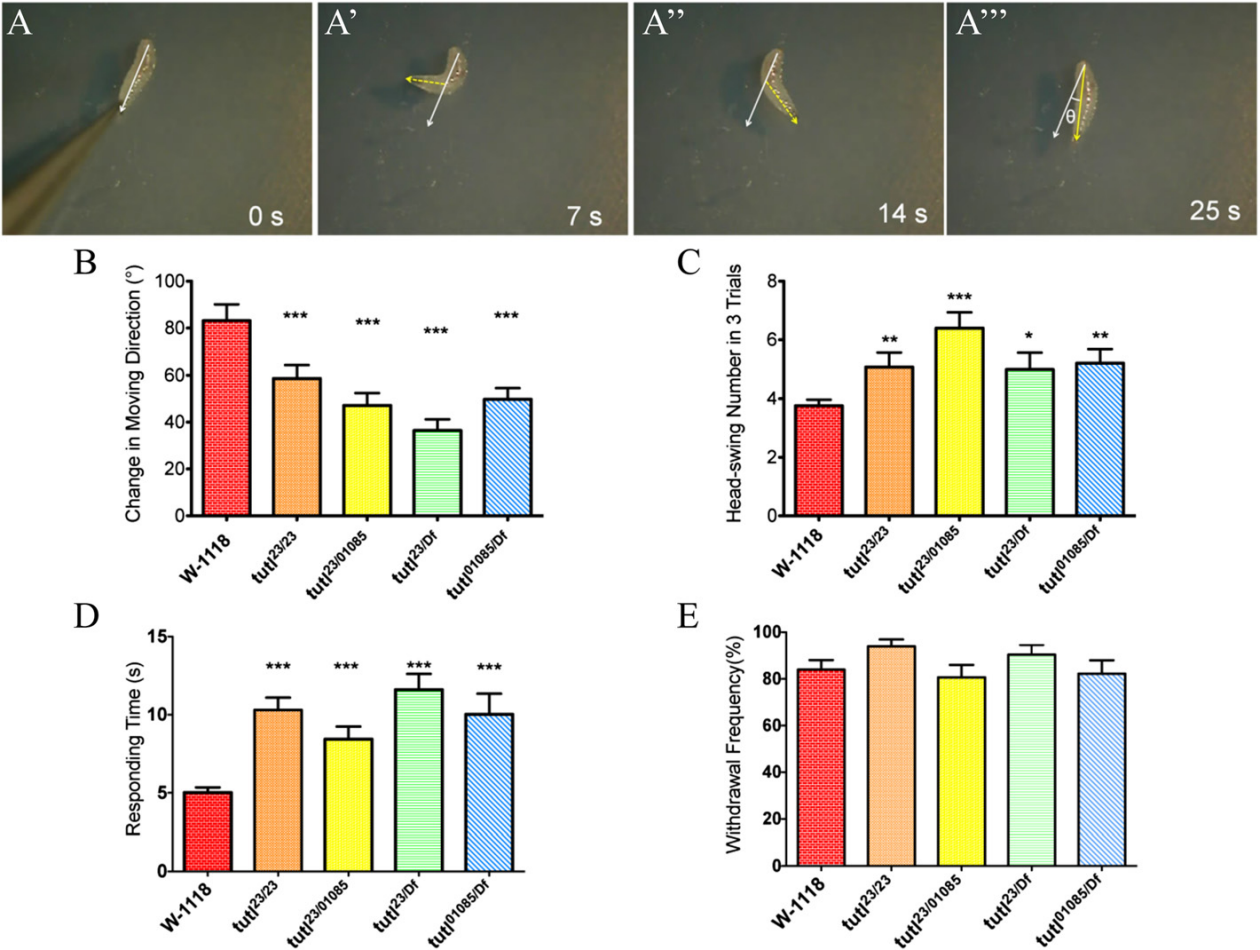
***Tutl *mutations affected larval navigational pattern in response to gentle touch.** (**A**-**A**”’) Time course of navigational pattern of *tutl*^*23/01085*^ mutant larvae in response to gentle touch at anterior segments. (**B**) *tutl* mutant larvae showed severe defects in adjusting moving direction. The performance of each genotype of *tutl* mutant larvae was compared to that of wild type. ^***^p<0.005, t-test. Number of larvae tested: *W-1118*, n=21; *tutl*^*23/23*^, n=20; *tutl*^*23/01085*^, n=20; *tutl*^*23/Df*^ , n=17; *tutl*^*01085/Df*^ , n=15. (**C**) *tutl* mutant larvae displayed higher numbers of exploratory head swings in response to gentle touch. ^*^p < 0.05, ^**^p < 0.01, ^***^p<0.005, t-test. (**D**) *tutl* mutant larvae took longer time to select a new moving direction after gentle touch. ^***^p<0.005, t-test. (E) *tutl* mutant larvae displayed normal withdrawal response after gentle touch. p>0.1, one-way ANOVA test. Error bars represent SEM.

Compared to wild type (Figure 1A-A”’), we found that many *tutl* homozygous or transheterozygous mutant larvae showed defects in changing their forward moving direction after gentle touch at anterior segments (Figure 4A-A”’), while heterozygous larvae displayed normal navigational pattern (data not shown). Data quantitation showed that *tutl* mutations caused a significant decrease in directional change in response to tactile stimuli (Figure 4B). In addition, prior to reorientation of forward movement, *tutl* mutant larvae performed more exploratory head swings (Figure 4A’, 4A” and 4C). It also took much longer time for *tutl* mutant larvae to select a new direction of forward movement after tactile stimuli (Figure 4A-A”’ and 4D).

To determine if the above defects were due to a reduction in sensation of gentle touch, we examined withdrawal response, which occurs before selection of new moving direction after gentle touch. Surprisingly, we found that *tutl* mutant larvae, like wild type, displayed normal withdrawal response after gentle touch (Figure 4E). This result indicates that *tutl* mutant larvae could still sense gentle touch.

### *Tutl* mutations did not affect general locomotion patterns

**Figure 5:**
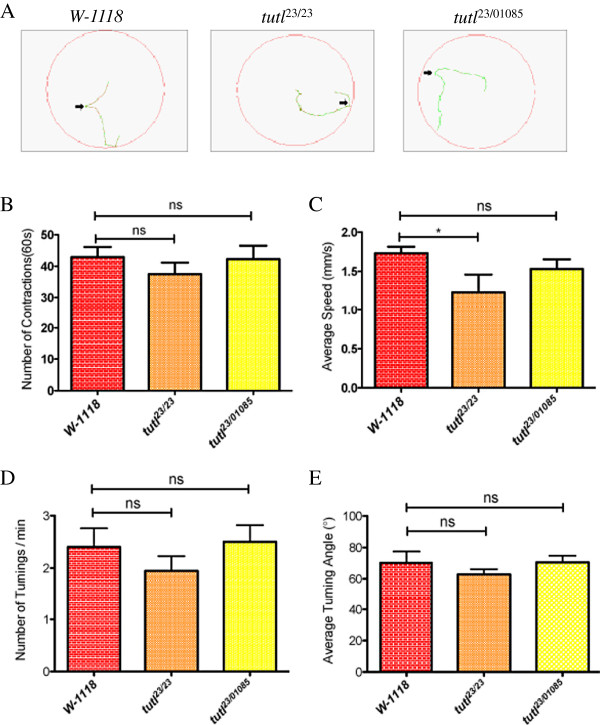
***Tutl *mutant larvae displayed normal locomotion pattern.** (**A**) Free movements of 3^rd^-instar larvae for three minutes on the surface of 2.5% agarose in 100 mm petri dish were recorded. Green: movements with a speed<1.5 mm/sec; red: movements with a speed>1.5 mm/sec. Arrows indicate examples of turning. (**B**) Number of contraction waves during a 60-second period were counted (n=10 for each genotype). No significant difference was observed between *tutl* mutant and *w-1118* larvae (p>0.1 for both t-test and one-way ANOVA test). “ns” indicates no significant difference. (**C**) Average speed during 3-min free larval locomotion was measured. No significant difference in average speed was observed between *tutl*^23/01085^ and *W-1118* larvae (p>0.1, t-test). Average speed of *tutl*^*23/23*^ larvae was slower than that of *W-1118* (^*^p<0.05, t-test). (**D**) Number of turnings during 3-min free larval locomotion was analyzed. No significant difference was observed between *tutl* mutant and *w-1118* larvae (p>0.1 for both t-test and one-way ANOVA test). (**E**) The change in moving direction after turning during 3-min free larval locomotion was measured. No significant difference was observed between *tutl* mutant and *w-1118* larvae (p>0.1 for both t-test and one-way ANOVA test). Error bars represent SEM.

We then examined if *tutl* mutations affect general locomotion pattern. Larval locomotion patterns in a stimulus-free condition were examined by using a digital video recording and analysis system (see Methods). Foraging larvae stereotypically alternate between long episodes of forward movement, and brief episodes of head swinging and reorientation [25]. During a 3-min period, we examined the path of movements (Figure 5A), number of contractions (Figure 5B), average speed (Figure 5C), number of turnings (Figure 5D), and average turning angles (Figure 5E). We found that compared to wild type, *tutl* mutant larvae displayed similar locomotion patterns. These results indicate that *tutl* mutations did not disrupt the general locomotor system, and *tutl* mutant larvae were capable of making a large-angle turn during reorientation.

### *Tutl* mutations did not affect larval phototaxis

**Figure 6:**
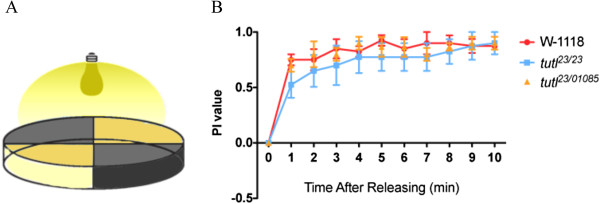
***Tutl *mutant larvae displayed normal phototaxis behaviors.** (**A**) A schematic diagram of the phototaxis assay. Briefly, the arena is divided into four quadrants, and two of which are covered with black paper. The arena is then illuminated with a light source from above. (**B**) The performance of larvae in phototaxis assay was examined (four trials). Performance index (PI) was estimated as follows: PI = (number of larvae in two dark quadrants - number of larvae in two bright quadrants) / (number of larvae in two dark quadrants + number of larvae in two bright quadrants). There were four trials for each genotype, n=20 per trial. p>0.05, one-way ANOVA test. Error bars represent SEM.

To determine if *tutl* mutations affect other types of sensorimotor behaviors, we examined the behaviors of *tutl* mutant larvae in response to light stimulation by performing the Darth Vader assay [26] (Figure 6A). Wild-type 3^rd^-instar foraging larvae exhibit strong preference for dark area [27] (Figure 6B). No significant difference in phototaxis behavior was observed between wild-type and *tutl* mutant larvae (Figure 6B). Like wild type, *tutl* mutant larvae were able to coordinate their movements towards dark area (Figure 6B).

### Cell-type-specific expression of a *tutl* transgene rescued navigational pattern in *tutl* mutants in response to gentle touch

Above results indicate a specific role for *tutl* in the control of navigational pattern after gentle touch, which presents an excellent starting point for genetic dissection of molecular networks and neuronal circuitry involved. Previous studies show that *tutl* is exclusively expressed in the nervous system [22,28,29]. To identify neurons in which *tutl* functions to regulate directional change, we performed rescue experiments.

A set of cell-type-specific GAL4 drivers were used to restore the expression of *tutl* in different types of neurons in the nervous system (Table 1). Pan-neuronal expression of a *tutl* transgene under control of the C155-GAL4 driver completely rescued the navigational phenotype (Table 1). Expression of *tutl* in amyloid–positive neurons under control of the Appl-GAL4 driver, or in cholinergic neurons under control of the Cha-GAL4 driver, also substantially rescued the phenotype (Table 1).

**Table 1:** **Transgene rescue of the navigational phenotype by expressing a UAS-*tutl *transgene under control of cell-type-specific GAL4 drivers**

**GAL4 driver**	**Expressing pattern**	**Change in moving direction (°) (Mean±SEM)**	**Rescue effect**
C155-GAL4	All post-mitotic neurons	93.8±5.5	Y^a^
Appl-GAL4	Many PNS and CNS neurons	107.0±5.3	Y
Cha-GAL4	Cholinergic neurons in PNS and CNS	74.0±5.2	Y
OK371-GAL4	Glutamatergic neurons (motor neurons and neuronal clusters in the brain)	_^c^	N^b^
Ddc-GAL4	Dopaminergic and serotonergic neurons	33.4±3.5	N
RN2-GAL4	RP2, aCC and pCC	38.4±3.7	N
G11-1-GAL4	Embryonic PNS	49.6±5.6	N
ftz.ng-GAL4	Subsets of neurons	40.0±4.4	N
D42-GAL4	Motor neurons and PNS neurons	76.4±8.7	N
TrpA1-GAL4	CNS neurons expressing TrpA1 gene	51.1±6.9	N
5-HTR1B-GAL4	Neurons expressing serotonin receptor 1B	46.9±4.5	N
C81-GAL4	CNS neurons with diffuse expression throughout brain lobes	45.8±4.7	N
SN(5-40)-GAL4	All sensory neurons	56.6±5.1	N
NompC-GAL4	Class I , bd neurons and chordotonal organs	47.7±4.0	N
iav-GAL4	Chordotonal organs	46.2±3.5	N
Pain-GAL4	md neurons, chordotonal organs and some CNS neurons	52.1±4.7	N
ppk1.9-GAL4	Class IV da neurons	51.2±5.3	N

3^rd^-instar *tutl*^*23*^/*tutl*^*23*^ mutant larvae, in which UAS-*tutl* was under control of different cell-type-specific GAL4 drivers, were examined for navigational pattern in response to tactile stimuli. Their performance was compared to that of *tutl*^*23*^/*tutl*^*23*^ mutant larvae that only carry GAL4.

^a^ “Y” indicates significant rescue (p<0.05).

^b^ “N” indicates no significant rescue.

^c^ Expression of *tutl* under control of Ok371-GAL4 in *tutl* mutant larvae caused a failure for larvae to resume forward movement after gentle touch.

Neurons co-expressing Appl-GAL4 and Cha-GAL4 are broadly distributed in the peripheral (PNS) and CNS (data not shown), suggesting that proper navigation decision after gentle touch requires the function of *tutl* in both sensory and central compartments. Consistently, we found that expression of *tutl* under control of the SN (5–40)-GAL4 driver, which drives gene expression in all PNS sensory neurons but not in CNS neurons [19], was not sufficient to rescue the phenotype (Table 1). That Cha-GAL4 is not expressed in motor neurons (data not shown), together with a failure of rescue with the drivers (e.g. ftz.ng-GAL4 and OK371-GAL4 [30]) for motor-neuron expression (Table 1), argue against a requirement of *tutl* in motor neurons.

### *Tutl* is required at larval stage

**Figure 7:**
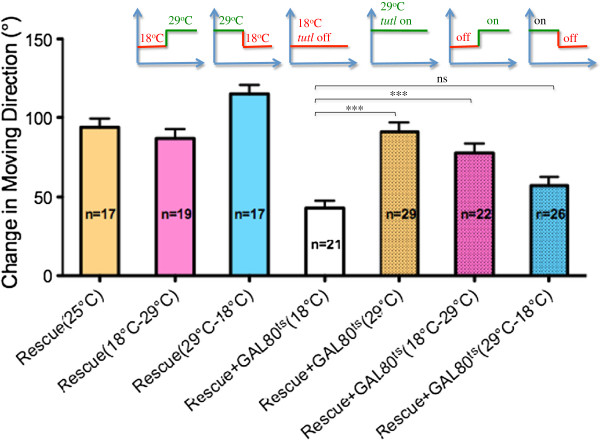
***Tutl *is required at larval stage after the completion of embryonic development.** “Rescue” refers to the group of *tutl*^*23/23*^ mutant larvae that carry UAS-*tutl* transgene under control of the pan-neuronal-specific driver C155-GAL4. “Rescue+GAL80^ts^” refers to the group of “Rescue” larvae that also carry a temperature-sensitive GAL80 (GAL80^ts^) under control of *tubulin* promoter. GAL80 is active at 18°C, allowing it to inhibit GAL4 and thus turning off the expression of *tutl* transgene. At 29°C, GAL4 is inactivated, allowing GAL4 to turn on the expression of *tutl* transgene. Number in each bar indicates the number of larvae tested. A shift of temperature thus allowed us to turn on or turn off *tutl* transgene expression after the completion of embryonic development. ^***^p<0.005, “ns” indicates p>0.05, t-test. Error bars represent SEM.

To determine the temporal requirement of *tutl*, we used the TARGET system [14] to manipulate the expression of *tutl* at embryonic or larval stages. We found that turning on the expression of a *tutl* transgene immediately after the completion of embryonic development was sufficient to rescue the navigational phenotype (Figure 7). Conversely, turning off the expression of *tutl* transgene at larval stage immediately after the completion of embryonic development, caused a failure in phenotypic rescue (Figure 7). Together, these results suggest strongly that Tutl acts at larval stage to modulate navigational pattern in response to gentle touch.

### A small subset of *tutl*-positive neurons were involved in modulating navigational pattern in response to tactile stimuli

There are a large number of *tutl*-positive neurons co-expressing Appl-GAL4 and Cha-GAL4, which are widely distributed in the nervous system (data not shown). Such a large number of *tutl*-positive neurons are likely involved in regulating many different behaviors. To gain insights into neuronal circuitry underlying the control of directional change, it is necessary to identify *tutl*-positive neurons that are specifically involved in regulating navigational behaviors.

**Figure 8:**
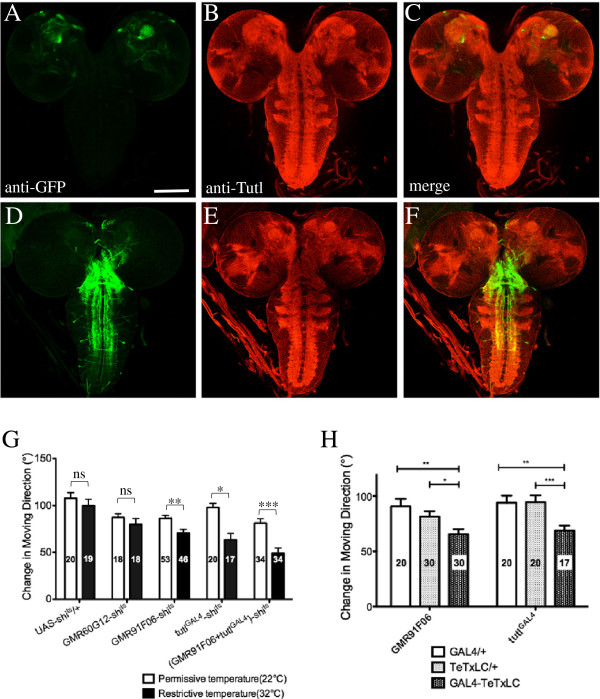
**Blockage of synaptic transmission in a subset of *tutl*-positive neurons significantly affected navigational pattern in response to tactile stimuli.** (**A**-**C**) Larvae carrying GMR91F06-GAL4 and UAS-CD4-tdGFP were double-stained with anti-GFP (green) and anti-Tutl antibody (red). Note Tutl protein is widely expressed in the nervous system and is predominantly localized to the neuropils of the CNS (**B** and **C**). (**D**-**F**) Larvae carrying *tutl*-GAL4 and UAS-CD4-tdGFP were double-stained with anti-GFP (green) and anti-Tutl antibody (red). Scale bars: 50 μm. (**G**) Navigational behaviors of 3^rd^-instar larvae in which UAS-*shi*^ts^ was driven by GMR91F06-GAL4, *tutl*-GAL4, or GMR60G12-GAL4. The performance of larvae at restrictive temperature (32°C, black bars) was compared to that of same-genotype larvae at permissive temperature (22°C, white bars). ^*^p < 0.05, ^**^p < 0.01, ^***^p<0.005, “ns” indicates p>0.05, t-test. Number in each bar indicates the number of larvae tested in the experiments. (**H**) Navigational pattern of larvae in which UAS-TeTxLC was driven by GMR91F06-GAL4 or *tutl*-GAL4, was examined. ^*^p < 0.05, ^**^p < 0.01, ^***^p<0.005, “ns” indicates p>0.05, t-test. Error bars represent SEM.

One way to approach this is to examine the effects of silencing subgroups of *tutl*-positive neurons on navigational pattern in response to gentle touch. This approach involves the expression of *shi*^ts^ in subgroups of *tutl*-positive neurons at restrictive temperature to block their synaptic transmission (see Methods). We tested a set of GAL4 drivers that are expressed in different subgroups of *tutl*-positive neurons. We found that expression of *shi*^ts^ under control of GMR91F06-GAL4 or *tutl-*GAL4, significantly affected navigation decision in response to tactile stimuli (Figure 8).

GMR91F06-GAL4 was generated by placing GAL4 under control of an enhancer element in the *tutl* gene [31], and is expressed in a small subset of *tutl*-positive neurons exclusively in the CNS (Figure 8A-C). *tutl-*GAL4 was generated by inserting GAL4 into the *tutl* gene [28]. *tutl-*GAL4 is expressed in a subset of *tutl*-positive neurons including class III da neurons in the PNS (data not shown) and a subset of neurons in the CNS (Figure 8D-F). Blocking synaptic transmission in GMR91F06-GAL4-positive neurons or *tutl-*GAL4-positive neurons by shifting from permissive temperature to restrictive temperature, caused a significant decrease in directional change after tactile stimuli (Figure 8G). Whereas expression of *shi*^ts^ under control of GMR60G12-GAL4, a driver in which GAL4 is driven by an enhancer element in the *Appl* gene [31], did not affect navigational pattern (Figure 8G). Interestingly, while larvae in which GMR91F06-GAL4-positive neurons or *tutl-*GAL4-positive neurons were silenced, displayed significant changes in navigational pattern, they were still able to withdraw from the stimuli (data not shown). Since withdrawal response is the first response after gentle touch before larvae reorient, this result is consistent with a role for these *tutl*-positive neurons in central information processing, but not in sensation of gentle touch.

We then examined the effects of blocking synaptic transmission simultaneously in both *tutl-*GAL4-positive neurons and GMR91F06-GAL4-positive neurons. We found that silencing both types of neurons simultaneously generated an even greater effect (Figure 8G). This suggests that *tutl-*GAL4-positive neurons and GMR91F06-GAL4-positive neurons function together to modulate navigational pattern in response to tactile stimuli.

We also took an alternative approach to block synaptic transmission in *tutl*-positive neurons by expressing tetanus toxin light chain (TeTxLC), which blocks evoked synaptic transmission by cleaving synaptic vesicle protein synaptobrevin [15]. UAS-TeTxLC was expressed under control of GMR91F06-GAL4 or *tutl-*GAL4. Consistent with the results from circuit breaking analysis with *shi*^ts^ (Figure 8G), we found that blockage of synaptic transmission in GMR91F06-GAL4-positive neurons or *tutl-*GAL4-positive neurons with TeTxLC, also significantly affected navigational pattern after tactile stimuli (Figure 8H).

Together, above results suggest strongly that small subset of *tutl*-postive neurons defined by *tutl-*GAL4 and GMR91F06-GAL4 are required specifically in neuronal circuitry that modulate navigational pattern in response to tactile stimuli.

## Discussion

In this study, we investigated the control of directional change in response to gentle touch in Drosophila. We showed that navigational pattern was affected by the intensity of stimuli, but not by gender difference. Consistently, reducing sensory inputs by blocking inputs from chordotonal organs or class IV da neurons significantly affected navigational pattern in response to light touch. Our genetic analysis revealed a role for the *tutl* gene in the control of navigational behaviors. Circuit analysis identified a small subset of *tutl*-positive neurons that are specifically required for modulating directional change in response to gentle touch.

Consistent with the correlation between stimulus intensity and the extent of directional change, our results showed that reducing sensory inputs by blocking synaptic transmission in chordotonal organs or class IV da neurons, led to a significant decrease in directional change in response to light touch (i.e. 1 mN). The role of chordotonal organs in larval mechanosensation has been reported by several previous studies. For instance, several genes whose mutations caused defects in response to tactile stimuli [7], were shown to be expressed and functionally required in chordotonal neurons [32,33]. Moreover, disrupting the structural integrity of chordotonal organs [17], or disrupting the connection of chordotonal neurons with their post-synaptic targets in the CNS [34], caused a decrease in sensitivity to touch and vibration, respectively.

Our results indicate that in addition to a role in mechanical and thermal nociception [16,20], class IV da neurons also mediate mechanosensation in response to light touch. Previous studies show that larvae in which class IV neurons carry mutations in genes encoding mechanotransducers such as *pain*, *pickpocket* and *piezo*, displayed defects in mechanical nociception, but showed normal sensitivity to gentle touch [11,16,20]. Together, these studies suggest that class IV da neurons mediate mechanotransduction in response to gentle touch by employing a mechanism different from that in mechanical nociception. Further studies are needed to elucidate the exact mechanism by which class IV da neurons mediate mechanotransduction in response to gentle touch.

Interestingly, we found that when the intensity of tactile stimuli was increased from 1 mN to 7 mN, blockage of sensory inputs from chordotonal organs or class IV da neurons did not affect withdrawal response nor the pattern of directional change. One possible explanation is that stronger stimulus intensity may significantly increase mechanoreceptor currents in other types of mechanosensitive neurons, for instance, external mechanoreceptive sense organs inserted in the cuticle, which may compensate for loss of inputs from chordotonal organs or class IV da neurons leading to normal navigational behaviors.

Behavioral analysis of *tutl* mutant larvae reveals an interesting phenotype in the adjustment of moving direction after gentle touch. While *tutl* mutant larvae were able to withdraw from tactile stimuli similarly as wild-type larvae, they displayed severe defects in adjusting moving direction after gentle touch. That *tutl* mutant larvae were capable of making large-angle turns during the course of free movements, argues against a general defect in the sensorimotor system. Consistent with this notion, we found that *tutl* mutant larvae displayed normal phototaxis behaviors. These results suggest strongly that mutations in the *tutl* gene specifically affect the circuits that modulate the changes in moving direction in response to gentle touch.

Our results from transgene rescue indicate that Tutl is required exclusively in post-mitotic neurons at larval stage after the completion of embryonic development, which is consistent with neuronal-specific expression pattern of endogenous Tutl. Restoration of *tutl* expression in Appl-positive neurons or cholinergic neurons also substantially rescued the navigational phenotype. Consistently, triple labeling highlighted a large population of cholinergic neurons positive for both Tutl and Appl in the nervous systems (data not shown). Appl-positive neurons are distributed broadly in the larval nervous system, including most of sensory neurons in the PNS and interneurons in the CNS [35]. Mutations in the *Appl* gene caused mild defects in locomotor reactivity [36], suggesting a role for Appl-positive neurons in the control of fly locomotion. Similarly, the larval cholinergic system includes many sensory neurons (e.g. chordotonal and da neurons) and a large group of interneurons in the CNS [37,38]. Blockage of synaptic transmission in all cholinergic neurons caused paralysis [13], while silencing communication between random cholinergic neurons caused several types of locomotor defects such as sluggish movement, failure in initiation or maintenance of locomotion, uncoordinated movement, and arrest of locomotion [39]. Taken together, those studies suggest that Appl-positive cholinergic neurons may form a functional circuit consisting of sensory neurons in the PNS and interneurons in the CNS, which controls larval sensorimotor decision making.

Tutl may function in Appl-positive cholinergic neurons in both PNS and CNS for proper navigational pattern in response to gentle touch. Consistent with a role for Tutl in sensory neurons, previous studies showed that mutations in the *tutl* gene caused defects in dendritic patterning of class I, II, III and IV da neurons in the PNS [29,40]. Two lines of evidence support that Tutl also plays a role in the CNS for adjusting moving direction after gentle touch. First, expression of *tutl* transgene in all peripheral sensory neurons was not sufficient for rescuing the navigational phenotype. And second, blockage of synaptic transmission in a small subset of *tutl*-positive neurons in the CNS significantly affected navigational pattern in response to gentle touch. These *tutl*-positive CNS neurons may function in the circuits that integrate and process information from tactile stimuli, thus allow animals to adjust their moving direction properly.

Tutl may play a role during the development of larval nervous system for hardwiring of neuronal circuits that are specifically involved in directional adjustment in response to gentle touch. Such a role for Tutl in circuit development is supported by several recent studies. For instance, our recent studies show that Tutl is involved in regulating axonal tiling and dendrite self-avoidance [28,29], two important cellular mechanisms that pattern neuronal circuitry during development [41]. It is also suggested that Tutl play a role in regulating axonal pathfinding at embryonic stage [42].

Alternatively or additionally, Tutl may also play a role in modulating the activity of the circuits for adjusting moving direction in response to gentle touch. In vitro analysis shows that Tutl can function as a homophilic cell adhesion molecule [28]. Many homophilic cell adhesion molecules have been shown to mediate synaptic function [43,44]. For instance, the well-known homophilic cell adhesion molecule Fasciclin II (FasII), and its mammalian homolog NCAM, have been implicated in regulating synaptic plasticity [45-47]. In this context, it is also worth noting that interfering with the function of Dasm1, the mouse homolog of Tutl, prevents synapse maturation in cultured hippocampal neurons [24]. Further studies are needed to elucidate the exact action of Tutl in the development and/or function of the circuits that control navigational pattern in response to gentle touch.

## Conclusion

Our study identifies Tutl and a small subset of CNS neurons in modulating directional change in response to gentle touch. The function of mammalian homologs of Tutl (i.e. Dasm1 in mice and IgSF9 in humans) is still unknown. Given high homology between Tutl and its mammalian homologs [22-24], it is possible that Dasm1/IgSF9 play a similar role in directional change after mechanical stimulation in mammals. The implication of Tutl and a small subset of CNS neurons in the control of directional change after gentle touch, presents an excellent starting point for further dissection of underlying molecular networks and neuronal circuitry.

## Methods

### Genetics

Flies were reared in plastic vials containing standard fly food or in grape juice plates at 25°C with ~50% humidity. Grape juice plates were prepared by mixing 30 g agar, 30 g sugar and 354 ml grape juice in 1.2L ddH_2_O. Flies for behavioral tests were kept in incubators with 12h light/dark cycle.

The following fly stocks were obtained from the Bloomington Stock Center: Appl-GAL4 (BL#30546), Cha-GAL4 (BL#6798), OK371-GAL4(BL#26160), Ddc-GAL4(BL#7009), RN2-GAL4(BL#7472), G11-1-GAL4 (BL#7030), ftz.ng-GAL4(BL#8767), D42-GAL4(BL#8816), TrpA1-GAL4(BL#27593), 5-HTR1B-GAL4(BL#27637), C81GAL4(BL#3738), Pain-GAL4(BL#27894), GMR91F06(BL#47170), GMR60G12 (BL#45360), tubP-GAL80^ts^(BL#7017), UAS-mCD8-GFP (BL#5136), UAS-CD4-tdGFP(BL#35838), UAS-TeTxLC(BL#28838), *pain*^*1*^(BL#27895), *pain*^*3*^(BL#31432), *tutl*^*01085*^(BL#10979). *tutl*^*23*^, *tutl*-GAL4, and UAS-*tutl*, were generated in our previous studies [28,29]. pBac[WH] [f03313] and pBac[WH]CG16857[f02225] were used to generate *tutl*^*Df*^, which removes *tutl* and *CG16857* by using the FLP/FRT-based strategy [48].

For cell-type-specific transgene rescue, genetic crosses were performed to generate *tutl*^*23*^ homozygous mutant larvae carrying UAS-*tutl* and GAL4 driver. Their navigational pattern was then compared to that in *tutl*^*23*^ homozygous mutant larvae carrying only GAL4 driver. For temporal control of UAS-*tutl* expression in *tutl* mutants using the TARGET system [14], larvae were raised with 12 hr light/dark cycle and moved between 18°C and 29°C incubators to turn on or turn off *tutl* transgene expression in *tutl*^*23*^ mutants. For circuit breaking analysis, flies carrying GAL4 drivers were crossed with UAS-*shi*^ts^ flies, and were raised at 22°C. Larval behaviors at permissive temperature (i.e. 22°C) or restrictive temperature (i.e. 32°C) were examined in a transparent box with precise temperature control (Kooland incubator).

### Gentle touch assay

3^rd^-instar larvae were collected and gently washed in ddH_2_O before transferred to 60 mm petri dish containing 2.5% agar substrate. Larvae were allowed for 3-min free locomotion prior to tactile stimuli. Gentle touch was applied to anterior segments of a larva at 25°C (22°C or 29°C for circuit breaking analysis). Filaments used for applying different stimulus intensities (i.e. 1 mN, 3 mN, 7 mN, 10 mN) were calibrated similarly as described previously [10]. Navigational pattern of each larva in response to tactile stimuli was tested four times during the course of forward movements. Larval navigational behaviors were recorded with a digital monochrome camera (LTC 0335, BOSCH), and analyzed using the MB-ruler software (MB-Software solutions).

### Mechanical nociception assay

Mechanical nociception assay was performed similarly as described previously [10,16]. Briefly, 3^rd^-instar larvae were stimulated with a 50 mN calibrated Von Frey filament. Noxious mechanical stimuli were delivered by rapidly touching the larva with the fiber at abdominal segments (i.e. four to six). A positive escape response was scored if at least one 360° revolution around the anterior/ posterior axis occurred in response to the stimuli. Each larva was tested only once. For each genotype, three trials (20–30 larvae per trial) were performed.

### Phototaxis (Darth Vader) assay

A slightly modified version of the Darth Vader assay was used [26]. Larvae were raised on grape juice plates with 1.25g/L β-carotene (Jamieson.). A 100 mm petri dish containing 2.5% agarose was divided into four quadrants, and two of which were covered by black paper (as shown in Figure 6A). The dish was illuminated from above with incandescent light (40W). All experiments were done at night in a dark room. After the release of larvae at the center of the plate, the number of larvae in each sector were counted at every 1-min interval for 10 minutes. A preference index (PI) was calculated as: PI = (number of larvae in two dark quadrants - number of larvae in two bright quadrants) / (number of larvae in two dark quadrants + number of larvae in two bright quadrants).

### Larval locomotion pattern

After 1-min adaptation time, free movements of 3^rd^-instar larvae on a 100 mm plate containing 2.5% agarose were recorded with a digital monochrome camera (LTC 0335, BOSCH) for 3 min at 25 images/sec, and analyzed with the Videotrack 3.1.1 software (ViewPoint, Life Sciences Inc.). Turnings are defined as >30° in directional change, followed by linear locomotion.

### Histology

Larval CNS and/or body wall were dissected in phosphate buffer (pH 7.2), fixed in 3.2% paraformaldehyde for 50 min, washed three times with PB-TX (0.5% Triton-X 100 in 1x PBS), and incubated with primary antibody in 10% normal goat serum at 4°C for three hours. Primary antibodies used were: mouse monoclonal anti-GFP (1:500 dilution) (Invitrogen/Molecular Probes), chick anti-GFP (1:500 dilution) (Abcam), and rabbit anti-Tutl polyclonal antibody (1:60,000 dilution). Following secondary antibodies were used: Alexa-488 dye-conjugated anti-mouse antibody (1:500 dilution), Alexa-568 dye-conjugated anti-rabbit antibody (1:500 dilution), or Alexa-647 dye-conjugated anti-mouse antibody (1:500 dilution) (Invitrogen/Molecular Probes). Images were captured using an Olympus FV1000 Confocal LSM microscope.

For generating anti-Tutl antibody, PCR fragments encoding the extracellular region of Tutl was subcloned into the pIB/Fc expression vector for producing Tutl-Fc fusion protein in S2 cells. Tutl-Fc fusion protein was purified using Protein A-conjugated Sepharose column, and used to raise antibodies in rabbits by using standard methods. Specificity of anti-Tutl antibody was confirmed by immunostaining showing absence of *tutl* staining in *tutl* mutant larvae (data not shown).

### Statistical analysis

Student’s t-test and/or ANOVA test were used for statistical analysis. A best-fit linear-regression analysis was used to determine the correlation between navigation decision and the intensity of stimuli. Statistical analysis was performed with Excel 2007 (Microsoft Corp) or GraphPad Prism 5.0 (GraphPad software).

## Competing interests

Authors declare that they have no competing interests.

## Authors’ contributions

YZ conducted most experiments, and was involved in writing the manuscript. SC generated the line (i.e. *tutl*^Df^) that deletes the entire *tutl* gene. WC prepared Tutl-Fc fusion protein and used it to generate anti-Tutl antibody in rabbit. YR supervised the project and wrote the manuscript. All authors read and approve the manuscript.
